# Enhanced *Botrytis cinerea* Resistance of Arabidopsis Plants Grown in Compost May Be Explained by Increased Expression of Defense-Related Genes, as Revealed by Microarray Analysis

**DOI:** 10.1371/journal.pone.0056075

**Published:** 2013-02-06

**Authors:** Guillem Segarra, Gabriel Santpere, Georgina Elena, Isabel Trillas

**Affiliations:** 1 Unitat de Fisiologia Vegetal, Departament de Biologia Vegetal, Facultat de Biologia, Universitat de Barcelona, Barcelona, Catalonia, Spain; 2 Institute of Evolutionary Biology (Universitat Pompeu Fabra-Consejo Superior de Investigaciones Científicas), Parc de Recerca Biomèdica de Barcelona, Barcelona, Catalonia, Spain; 3 Departament de Patologia Vegetal, Institut de Recerca i Tecnologia Agroalimentàries Cabrils, Cabrils, Catalonia, Spain; National Taiwan University, Taiwan

## Abstract

Composts are the products obtained after the aerobic degradation of different types of organic matter waste and can be used as substrates or substrate/soil amendments for plant cultivation. There is a small but increasing number of reports that suggest that foliar diseases may be reduced when using compost, rather than standard substrates, as growing medium. The purpose of this study was to examine the gene expression alteration produced by the compost to gain knowledge of the mechanisms involved in compost-induced systemic resistance. A compost from olive marc and olive tree leaves was able to induce resistance against *Botrytis cinerea* in Arabidopsis, unlike the standard substrate, perlite. Microarray analyses revealed that 178 genes were differently expressed, with a fold change cut-off of 1, of which 155 were up-regulated and 23 were down-regulated in compost-grown, as against perlite-grown plants. A functional enrichment study of up-regulated genes revealed that 38 Gene Ontology terms were significantly enriched. Response to stress, biotic stimulus, other organism, bacterium, fungus, chemical and abiotic stimulus, SA and ABA stimulus, oxidative stress, water, temperature and cold were significantly enriched, as were immune and defense responses, systemic acquired resistance, secondary metabolic process and oxireductase activity. Interestingly, *PR1* expression, which was equally enhanced by growing the plants in compost and by *B. cinerea* inoculation, was further boosted in compost-grown pathogen-inoculated plants. Compost triggered a plant response that shares similarities with both systemic acquired resistance and ABA-dependent/independent abiotic stress responses.

## Introduction

Modern agriculture relies on inputs obtained from outside the farming system, such as chemical fertilizers, pesticides and substrates [1]. Expanded perlite is widely used for growing plants instead of soil, along with other substrates like peat, vermiculite and coconut fiber. As these materials are usually very poor in nutrients and microorganisms, they are regarded as easy to work with, as nutrition is supplied by adding standardized chemical fertilizers, and are basically pathogen-free. However, they also lack beneficial and saprophytic micro-organisms and, due to the lack of competition, the occasional intrusion of a pathogen usually leads to the spread of the disease [2].

Composts are the products obtained after the aerobic degradation (composting) of several different types of organic matter waste that can be used as substrates or substrate/soil amendments. These products are rich in nutrients and micro-organisms and may improve plant growth and health, so reducing the use of agrochemicals [3]. In addition, they are a sustainable alternative to standard substrates such as organic peat or inorganic perlite [4]. Certain composts are described as suppressive of soil-borne pathogens, as against standard substrates that tend to favor them. This suppressive quality was described as a combination of effects, including the competition and antibiosis produced by micro-organisms, the degree of degradation of the organic matter and the presence of inhibiting compounds and pH, among other factors [5]. Furthermore, there are a small but growing number of reports suggesting that foliar diseases are reduced when compost is used as a growing medium. Since the compost is not in direct contact with the pathogen, plant-mediated mechanisms appear to be the most suitable explanation. A common reaction of plants to biotic and abiotic stresses is the enhancement of basal resistance, which is often called induced resistance. The two archetypal cases of induced resistance are systemic acquired resistance (SAR) and induced systemic resistance (ISR). In SAR [6], the attack of a pathogen triggers defense responses, a local signal travels systemically and the entire plant increases its resistance to future attacks from various pathogens. SAR requires salicylic acid (SA) [7] and is related to the induction of pathogenesis-related (PR) proteins [8]. ISR is triggered by the inoculation of the plant with certain beneficial micro-organisms; the plant is stimulated to respond more quickly and intensely when the plant is attacked by a pathogen, but no gene expression changes are detected prior to pathogen infection [9]–[11]. ISR is dependent on jasmonic acid (JA) and ethylene (ET) [12]. It has been claimed that foliar disease reduction by composts is mediated by induced resistance [13], [14]. There are a small number of reports in the literature on compost-induced resistance. As several plant species and pathogens were used in these studies, the results are difficult to compare and are not always consistent. The first report on Arabidopsis by Zhang *et al.* [14] described compost-induced resistance that involved the strengthening of resistance responses after infection rather than their direct activation, as observed in beta-D-glucuronidase (GUS) activity driven by a *PR2* (beta-1,3-glucanase) gene promoter in transgenic compost-grown Arabidopsis plants. In contrast, Vallad *et al.* [13] described compost-induced resistance that was not operative in *npr1* Arabidopsis plants and was associated with increases in *PR1* and *PR2* induced by the compost itself, even though the effect of a subsequent challenge from the pathogen on gene expression was not studied. In addition, compost extracts applied as root treatments enhanced not only the expression of the pathogenesis-related genes CABPR1, CABGLU, CAChi2, CaPR-4, CAPO1 and CaPR-10 in pepper and PR1-1a, PR-2, PR-3 and APOX in cucumber, but also the activity of beta-1,3-glucanase, chitinase and peroxidase and the generation of hydrogen peroxide in pepper and cucumber under pathogen-inoculated conditions, but not under pathogen-free conditions [15].

The importance of the role of abscisic acid (ABA) and abiotic stress in plant pathogen interactions is gaining recognition and novel findings suggest crosstalk between their signaling pathways [16]. It is interesting to note that the salinity level of certain composts used as substrate correlated with the level of *Botrytis cinerea* resistance in cucumber plants [17]. Low temperature and dehydration are adverse environmental conditions that affect plant growth and productivity. Many genes that respond to both stresses at the transcriptional level have been described. Their gene products are thought to function in stress tolerance and response, even though these stresses are quite different [18]. Abiotic stress signal transduction pathways from signal perception to gene expression involve different *cis* and *trans*-acting elements. The basic leucine zipper factors, AREB/ABF and MYC/MYB proteins, activate the major ABA-dependent stress response through their corresponding *cis*-acting elements (ABREs, MYCRS and MYBRS, respectively). The DREB (drought responsive element binding) proteins activate the stress response through their *cis*-acting elements (DREs), in an ABA-independent manner. NAC play a role in both ABA-independent and ABA-dependent pathways. However, the ABA-dependent and ABA-independent pathways act in parallel and also interact, thereby providing added coordination between stress signals and ABA in the regulation of stress-responsive genes [19].

Preliminary results suggested that a compost from olive marc and olive tree leaves induced resistance against *Botrytis cinerea* in Arabidopsis. The purpose of this study was to unravel the gene expression alteration produced by the compost to gain knowledge about the mechanisms involved in compost-induced systemic resistance.

## Materials and Methods

### Plant material

Perlite and olive marc compost (OMC) were used as substrates. OMC was produced at the University of Seville (Spain), starting from a 1∶1.125 mixture of olive marc and olive tree leaves composted in piles for 19 weeks and then matured for one year. OMC pH was 7.9 and electrical conductivity was 1.0 dS/m. *Arabidopsis thaliana* Col-0 plants were grown in perlite trays in a growth chamber at 22°C, 70% RH and 8 h/day of 110 µmol m^−2^ s^−1^ PPFD. 17 days later, plants were transplanted to individual 60-mL pots containing either OMC or perlite and were randomly distributed in the growth chamber. The plants were watered with half-strength Hoagland solution (electrical conductivity was 1.7 dS m^−1^) every other day and maintained until they were 5 weeks old.

### Pathogen inoculation

*Botrytis cinerea* stored in silica gel was grown in a vegetable medium for 3 weeks at 22°C in a growth chamber with 16 h/day of 85 µmol m^−2^ s^−1^ PPFD. A vegetable medium was prepared by cooking 500 g of a commercial frozen mix of potato, carrot and beans in water. The boiled vegetables and cooking water were homogenized with a kitchen blender, the volume was brought to 1 L and 150 mL of the mixture plus 7.5 g of agar were used to prepare 500 mL of vegetable medium. Conidia were harvested in inoculation buffer containing 0.5 g L^−1^ glucose and 0.5 g L^−1^ KH_2_PO_4_ and conidia concentration was adjusted to 10^6^ conidia mL^−1^. One 3-µl drop of conidia suspension was applied to alternate mature leaves. Five plants grown in perlite and five plants grown in OMC were inoculated with the pathogen. The same numbers of plants were treated with buffer without conidia (control plants). After inoculation, plants were randomly distributed and kept at 100% RH. 3 days later, the plants were harvested for RNA extraction and the percentage of diseased leaves was recorded. The experiment was performed twice. Variance was homogeneous and thus data from the two experiments were combined. Significant differences were examined by analysis of variance (*P*<0.05).

### Chlorophyll fluorescence measurement

Chlorophyll fluorescence images were recorded by means of an Imaging-PAM, MICRO-version (Walz, Effeltrich, Germany), a chlorophyll fluorometer that provides all relevant chlorophyll fluorescence parameters, using the saturation pulse method. After 20 min of dark adaptation of the leaves, minimum fluorescence (Fo), maximum fluorescence (Fm) and maximum quantum yield of PSII photochemistry (Fv/Fm) (equivalent to (Fm–Fo)/Fm) were obtained [20]. Three replicates were used per experiment and the experiment was performed twice. Variance was homogeneous and thus data from the two experiments were combined. Significant differences were examined by analysis of variance (*P*<0.05). The two factors and their interaction were significant in the statistical analysis. For this reason a Duncan's multiple-range test was applied to detect the significant differences (*P*<0.05).

### Microarray

RNA was extracted from samples ground under liquid nitrogen by using SpeedTools Total RNA Extraction kit (Biotools, Madrid, Spain), according to the manufacturer's instructions. RNA quality and quantity were checked with a NanoDrop ND-1000 spectrophotometer and an Agilent 2100 Bioanalyzer. Samples were prepared according to the protocols outlined in the GeneChip Expression Analysis Technical Manual and hybridizations to the Affymetrix Arabidopsis Genome ATH1 Array were performed at the Functional Genomics Core Facility, Institute for Research in Biomedicine (Barcelona, Spain). Overall gene expression of plants grown in compost (3 biological replicates) was compared with expression of plants grown in perlite (2 biological replicates). The array data was standardized through the RMA (Robust Multichip Average) algorithm [21]; and differential expression analysis was performed by Limma (Linear Models for Microarray Data), which is a package for the R computing environment [22]. The microarray data were deposited at GEO (Gene Expression Omnibus) at the National Center for Biotechnology Information (NCBI) http://www.ncbi.nlm.nih.gov/geo/ with the accession number GSE42149.

### RT-qPCR

RNA extracted as mentioned above was converted to cDNA using oligo-dT20 primers, dNTPs and SuperScript III reverse transcriptase (Invitrogen, Alcobendas, Spain), according to the manufacturer's instructions. Quantitative PCR reactions took place in 384-well plates in an Applied Biosystems 7900HT Fast Real-Time PCR system, using Power SYBR Green PCR master mix (Applied Biosystems, Alcobendas, Spain), according to the manufacturer's instructions. Expression of *At1g15520*, *At1g19250*, *At4g19420*, *At2g30770*, *At2g43570*, *At1g45145*, *At5g59320*, *At3g61060*, *At1g73805* and *At2g14610* genes was corrected with the constitutively expressed reference gene *At1g13320* (*At1g13320fw*, TAA CGT GGC CAA AAT GAT GC; *At1g13320rev*, GTT CTC CAC AAC CGC TTG GT) [23]. Specific primers for all studied genes are reported in Table 1. Corrected expression levels were compared to those of control plants grown in perlite (set at 1). Significant differences were examined by analysis of variance (*P*<0.05). The two factors and their interaction were significant in the statistical analysis of all genes. For this reason a Duncan's multiple-range test was applied to detect the significant differences (*P*<0.05).

**Table 1:** Changes in gene expression estimated by microarray hybridization and by quantitative real-time reverse transcription-polymerase chain reaction (RT-qPCR).

*Arabidopsis thaliana* locus	Gene symbol	Primer F	Primer R	Fold change average*	
				Micro-array	RT-qPCR
*At5g59320*	*LTP3*	5′-CATTTCTGGTCTCAACCCAAG-3′	5′-CGACGTAAGCTTCCATTTCAC-3′	4.82	4.56
*At1g19250*	*FOM1*	5′-TGCTGTTCAGATCGGAGATTC-3′	5′-CGGTACACACAACCACGAAC-3′	3.92	2.87
*At1g15520*	*PDR12*	5′-TGATATATTCATGAAGGCGATGTC-3′	5′-TGCACAGACCTCAAGTCCTAAG-3′	3.05	2.27
*At2g43570*	*(CHI)*	5′-CATCTCCAAACGCGAAATC-3′	5′-GCTGGTCCATCAATTTCCTC-3′	2.67	2.07
*At2g14610*	*PR1*	5′-CTCGGAGCTACGCAGAACAA-3′	5′-TTCTCGCTAACCCACATGTTCA-3′	2.56	2.20
*At2g30770*	*CYP71A13*	5′-GATGTTGTGTTTGCTCCCTATG-3′	5′-TTGTTGGTGAGCAGATTGAGA-3′	2.18	3.14
*At1g73805*	*SARD1*	5′-TTGTTGTTAGAGATCATCGTGGA-3′	5′-CGAGAGGAGAGCTTCTTGTGA-3′	1.55	1.31
*At1g45145*	*TRX5*	5′-CGCCAATGAATCCAAGAAAC-3′	5′-TCTGCAAACACTGGTGCAAT-3′	1.55	1.10
*At3g61060*	*PP2-A13*	5′-ACTGGAATTGATGATCGGAGA-3′	5′-GAACATAAGCAGCTGACTGGAA-3′	−1.01	−0.84
*At4g19420*	*(PFP)*	5′-TCAAGATTAACTCCTGCAATGTGT-3′	5′-TGTTCTTTATCTGCCAAGAGTCA-3′	−1.09	−0.61

* Fold change expressed as log_2_ of expression in compost-grown plants minus log_2_ expression of plants grown in perlite.

Non-standard symbols appear in brackets.

### Functional enrichment

Functional enrichment of differentially expressed genes was analyzed by singular enrichment analysis (SEA) with the agriGO tool [24]. SEA analysis computes GO term enrichment in the selected set of genes by comparing it to the reference set (in this case, the Affymetrix ATH1 Genome Array). The statistical method used is the Fisher test. The Benjamini-Yekutieli method is used to do the multiple comparison correction.

### Transcription factor binding site enrichment

1,000 bp upstream promoter sequences of differentially expressed genes were analyzed by means of the Athena database and web interface, following the author's instructions [25]. Enrichment of transcription factor binding sites in the promoters was calculated by means of a hypergeometric probability distribution; *P*<0.05.

## Results

### Induced systemic resistance

**Figure 1:**
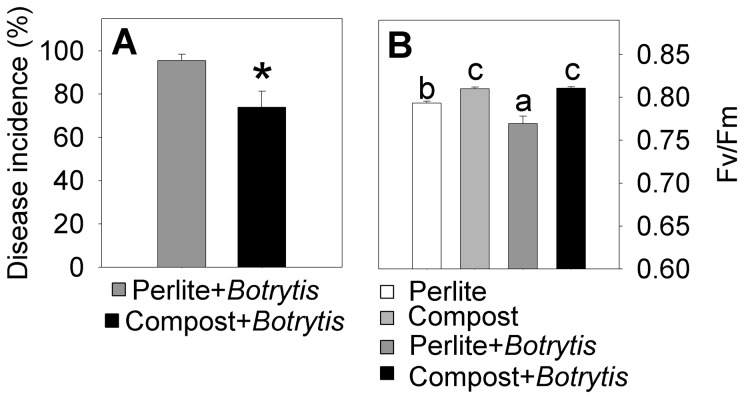
Percentage of infected leaves and Fv/Fm of Arabidopsis plants grown in perlite or compost. Percentage of infected leaves (A) and Fv/Fm (B) of Arabidopsis plants grown in perlite or compost 3 days after inoculation with *Botrytis cinerea* (3-µl drops of a 10^6^ conidia mL^−1^ suspension was applied to alternate mature leaves). Control plants were treated with buffer without conidia. Bars represent the mean ± standard error (n = 10 for percentage of infected leaves and n = 6 for Fv/Fm). An asterisk indicates significant differences (*P*<0.05) in the ANOVA test. Different letters indicate significant differences in a Duncan's multiple-range test, *P*<0.05.

After inoculation with the foliar pathogen *Botrytis cinerea*, Arabidopsis plants grown in compost had 22% fewer diseased leaves than plants grown in perlite (Fig. 1). As the pathogen was applied to the leaves and the substrate is only in contact with the roots, this disease reduction phenomenon associated with compost has to be systemic. In addition, plants grown in perlite and inoculated with *B. cinerea* had a smaller Fv/Fm than inoculated plants grown in compost, confirming that the plants grown in perlite were more affected by the disease (Fig. 1). Plants grown in perlite and inoculated with *B. cinerea* had lower Fv/Fm values than those of control perlite-grown plants. Interestingly, *B. cinerea* inoculation did not affect Fv/Fm in compost-grown plants.

### Differential gene expression revealed by microarray

After LIMMA treatment of our data and applying a FC cut-off of >1, we obtained 178 genes that were differently expressed (DE) in the two treatments, with a *P-*value of 0.05, of which 155 were up-regulated and 23 were down-regulated in compost-grown plants, as against perlite-grown ones (Table S1).

### GO term enrichment

**Figure 2:**
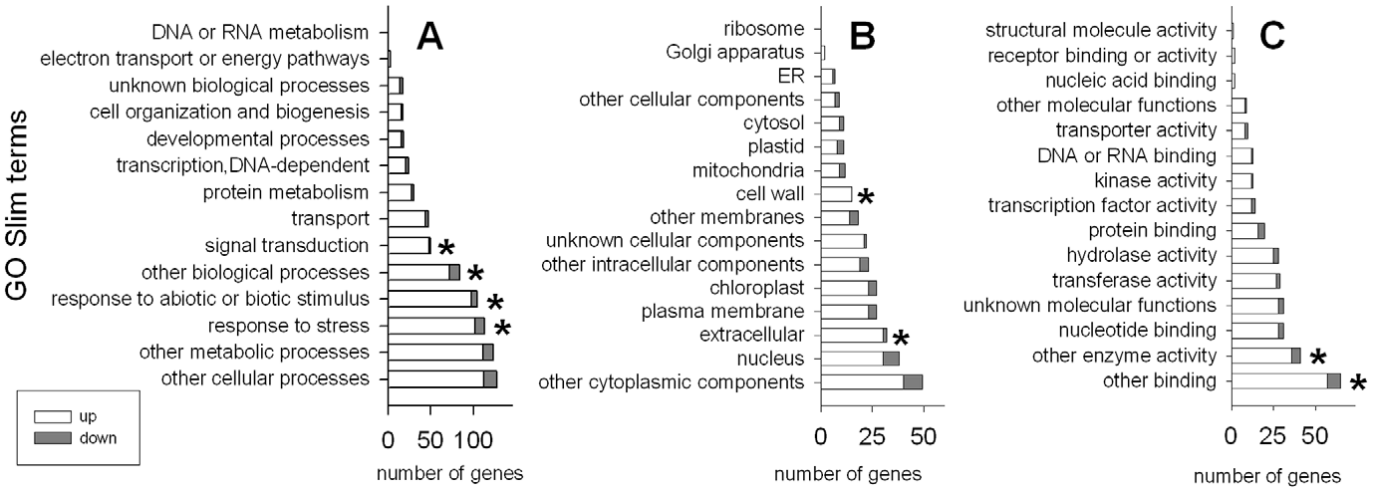
Gene Ontology Slim terms gene counts of the differentially expressed genes. Gene Ontology Slim terms gene counts for biological process (A), cellular component (B) and molecular function (C) of the differentially expressed genes (plants grown in compost vs. perlite). Up-regulated in white and down-regulated in gray. An asterisk indicates over-represented terms when comparing the abundance of the term in the pool of significant genes and in the whole microarray chip by Fisher's exact test (*P*<0.05, applying Bonferroni's correction).

Gene Ontology (GO) terms available at The Arabidopsis Information Resource (TAIR, www.arabidopsis.org) were assigned to the DE genes. Figure 2 shows the number of significant genes in the biological process, cellular component and molecular function categories, according to the GO Slim Classification for Plants. This classification was developed at TAIR to organize sets of genes according to broad GO ontology categories. Response to stress, response to abiotic or biotic stimulus, other biological processes and signal transduction were significantly over-represented terms in biological process (Fig. 2A), whereas extracellular and cell wall were over-represented in the cellular component (Fig. 2B); and other binding and enzyme activities, in molecular function (Fig. 2C).

**Figure 3:**
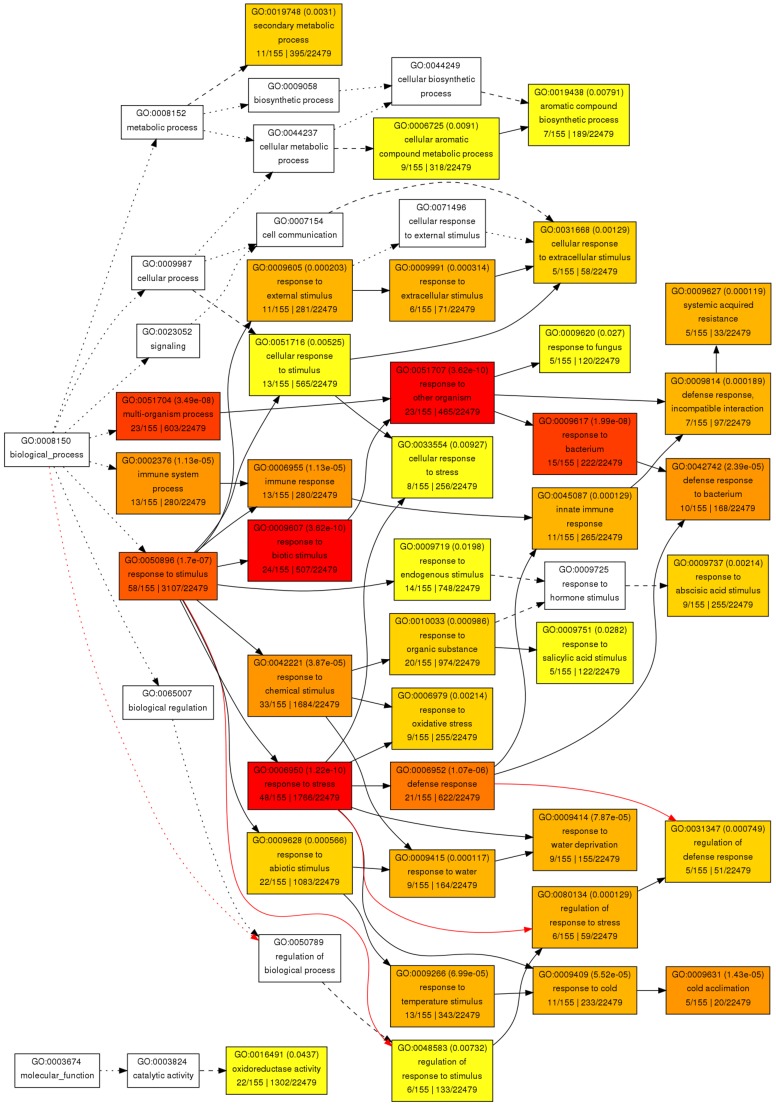
Hierarchical tree graph of over-represented GO terms in up-regulated genes by singular enrichment analysis generated by agriGO. Boxes in the graph show GO terms labeled by their GO ID, term definition and statistical information. The significant terms (adjusted *P*<0.05) are marked with color, while non-significant terms are shown as white boxes. The degree of color saturation of a box correlates positively with the enrichment level of the term. Solid, dashed and dotted lines represent two, one and zero enriched terms at both ends connected by the line, respectively. A red line indicates positive regulation. The rank direction of the graph runs from left to right.

A functional enrichment study of up-regulated genes revealed that 38 GO terms were significantly enriched (37 biological process terms and 1 molecular function term) (Fig. 3). Functional enrichment of down-regulated genes did not reveal any significant GO term enrichment. As can be seen in Figure 3, the most significantly enriched function was response to stress, followed by response to biotic stimulus, response to another organism, response to bacterium and multi-organism process. Response to fungus was also significantly enriched, but with a lower level of significance. Response to stimulus, chemical stimulus and abiotic stimulus and, in particular, response to SA and ABA stimulus, oxidative stress, water, temperature and cold were significantly enriched terms. Immune and defense responses and SAR were also enriched terms, as were secondary metabolic process and aromatic compound metabolic process. In addition, the molecular function's oxireductase activity was significantly enriched.

### Validation of microarray results

Gene expression from *LIPID TRANSFER PROTEIN 3 (LTP3), FLAVIN-DEPENDENT MONOOXYGENASE 1 (FMO1), PLEIOTROPIC DRUG RESISTANCE 12 (PDR12), CHITINASE (CHI), PATHOGENESIS-RELATED GENE 1 (PR1), CYTOCHROME P450 (CYP71A13), SAR DEFICIENT 1 (SARD1), THIOREDOXIN H-TYPE 5 (TRX5), PHLOEM PROTEIN 2-A13 (PP2-A13)* and *PECTINACETYLESTERASE FAMILY PROTEIN (PFP)* studied by RT-qPCR behaved similarly to the expression studied by microarray hybridization, thus supporting the microarray gene expression data (Table 1).

### Gene expression of Arabidopsis plants after *B. cinerea* inoculation

**Figure 4:**
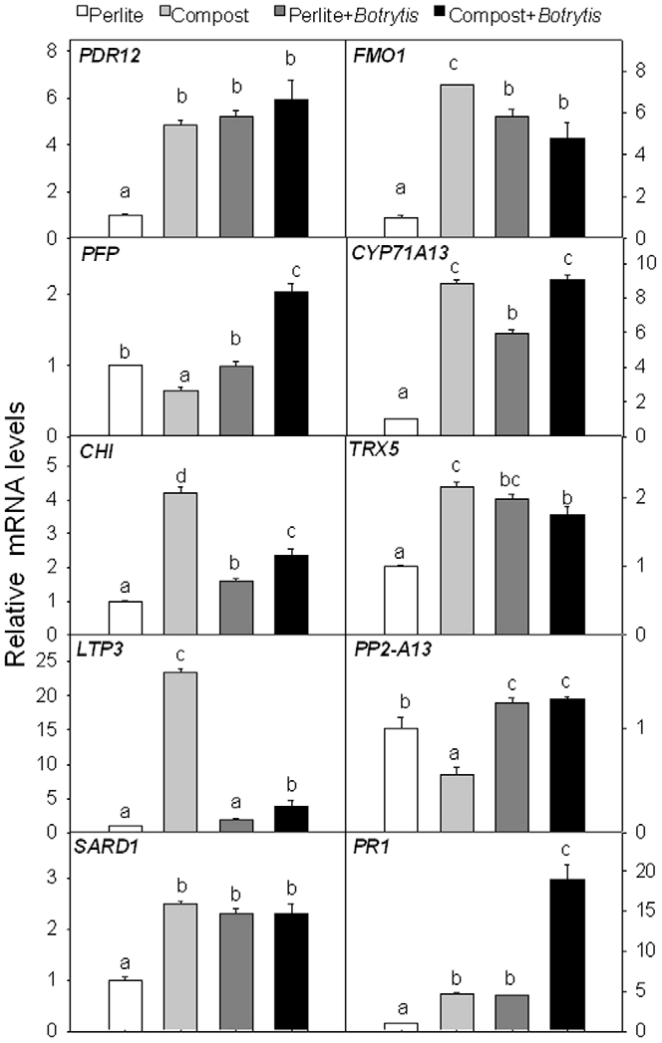
Expression levels of ten differently expressed genes in Arabidopsis plants inoculated with *B. cinerea*. Expression levels of *PLEIOTROPIC DRUG RESISTANCE 12 (PDR12)*, *FLAVIN-DEPENDENT MONOOXYGENASE 1 (FMO1)*, *PECTINACETYLESTERASE FAMILY PROTEIN (PFP)*, *CYTOCHROME P450 (CYP71A13)*, *CHITINASE (CHI)*, *THIOREDOXIN H-TYPE 5 (TRX5)*, *LIPID TRANSFER PROTEIN 3 (LTP3)*, *PHLOEM PROTEIN 2-A13 (PP2-A13)*, *SAR DEFICIENT 1 (SARD1)* and *PATHOGENESIS-RELATED GENE 1 (PR1)* in Arabidopsis Col-0 leaves of plants grown in either perlite or olive marc compost 3 days after inoculation with *Botrytis cinerea* (3-µl drops of a 10^6^ conidia mL^−1^ suspension was applied to alternate mature leaves). Control plants were treated with buffer without conidia. For reference, expression values of control plants grown in perlite are set at 1. Gene expression was corrected with the constitutively expressed reference gene *At1g13320*. Different letters indicate significant differences in a Duncan's multiple-range test, *P*<0.05; data shown are means ± SE, n = 3.

As can be seen in Fig. 4, *PDR12*, *FOM1*, *CYP71A13*, *CHI*, *TRX5*, *LTP3*, *SARD1* and *PR1* were expressed more in compost-grown than in perlite-grown plants, while *PFP* and *PP2-A13* expression decreased. *B. cinerea* inoculation of perlite-grown plants had an effect on increasing the gene expression of *PDR12*, *TRX5*, *SARD1* and *PR1* similar to the effect produced by using compost as substrate. On the other hand, *FMO1*, *CYP71A13* and *CHI* were induced less by *B. cinerea* than by compost. *LTP3* expression was not enhanced by *B. cinerea* in plants grown in perlite. Furthermore, in the case of *PFP* and *PP2-A13*, *B. cinerea*-inoculated plants had expressions equal to or higher than control plants, respectively, while compost down-regulated the expression. Interestingly, *PFP* and *PR1* expression was higher in plants grown in compost and afterwards inoculated with the pathogen than in plants grown in perlite and inoculated with *B. cinerea* or plants grown in compost alone. The *PDR12*, *FMO1*, *TRX5*, *PP2-A13* and *SARD1* expression of compost-grown plants inoculated with *B. cinerea* was not different from that of perlite-grown plants inoculated with *B. cinerea*. Concerning *PDR12*, *CYP71A13* and *SARD1* genes, the expression was similar in compost-grown and compost-grown pathogen-treated plants.

### Transcription factor binding site enrichment

Using the Athena database and web interface, we studied 1,000 bp upstream promoter sequences of differentially expressed genes and evaluated the enrichment of transcription factor binding sites (Table S2). ABRE-like binding site motif, CACGTG motif, Evening Element promoter motif, W-box promoter motif, Z-box promoter motif, CBF1 BS in *COR15A* and TATA-box motif were significantly enriched in the promoters of the up-regulated genes. No enrichment was found for the down-regulated genes.

## Discussion

Plants grown in compost were more resistant to *B. cinerea* than plants grown in perlite, as shown by fewer infected leaves and higher Fv/Fm.

The induction of resistance by growing plants on composts or compost-amended soils has been described in the literature [26], [27], though not in depth. In addition, some authors have described how compost water extracts also induce resistance to a foliar disease, when applied to plant roots [15]. Preliminary studies with OMC showed that altered gene expression in compost-grown plants, when compared to perlite-grown plants, could explain the enhanced resistance of Arabidopsis plants grown in compost. To gain insight into the induced resistance phenomenon, we performed microarray hybridization, which resulted in several differentially expressed genes in plants grown in compost vs perlite. To our knowledge, this is the first microarray experiment describing the effect that growing Arabidopsis in a compost substrate has on gene expression. It is interesting to note that, just by growing the plants in a different growth medium (perlite or compost), 178 genes were differently expressed with an FC cut-off of 1. Little is known about the effect that growing a plant on compost has on plant gene expression. Zhang *et al.* [14] reported that β-1,3-glucanase activity was low in cucumber plants grown in either compost or peat substrates, but when infected with *C. orbiculare* this activity was induced to significantly higher levels in plants grown in the compost mix than in plants grown in the peat mix. On the other hand, Vallad *et al.* [13] showed increases in *PR1* and *PR2* expression induced in Arabidopsis by the compost itself. Composts have different chemical, physical and microbiological properties, depending on the source of organic matter, composting process and degree of maturation, which may explain the different results obtained when using different composts. The OMC used in the present study is similar to that described in Segarra *et al.* [17], which induced resistance to *B. cinerea* in cucumber plants.

We found several PR genes up-regulated in compost-grown plants. PR proteins are induced upon infection with oomycetes, fungi, bacteria or viruses, or on insect attack, and possess antimicrobial activities *in vitro* through hydrolytic activities on cell walls, contact toxicity and perhaps an involvement in defense signaling [8]. Notably, the prominent PR1 proteins are often used as markers of the enhanced defensive state conferred by pathogen-induced SAR, but their biological activity has remained elusive [8]. In addition to the well-known *PATHOGENESIS-RELATED 1*, *BETA-1,3-GLUCANASE* and *THAUMATIN-LIKE* we found that *THIONIN 2.2* and *LIPID TRANSFER PROTEIN 3* were up-regulated in compost-grown plants. Thionins and lipid transfer proteins belong to the PR families 13 and 14, respectively, have broad *in vitro* antimicrobial activity and may act synergistically, leading to the permeabilization of cell membranes [28]. Furthermore, endogenous over-expression of three lipid transfer protein-like genes in *A. thaliana* resulted in enhanced tolerance to *B. cinerea* [29]. Interestingly, in our study, inoculation of perlite-grown plants enhanced *PATHOGENESIS-RELATED PROTEIN 1* expression as much as growing the plants in compost without the pathogen. Furthermore, inoculation of compost-grown plants led to an even higher expression. In the case of *LIPID TRANSFER PROTEIN 3*, this synergy was not found. As stated above, some composts on their own affect pathogenesis-related proteins, while others strengthen expression only after pathogen attack [13]–[15]. In addition, a putative *CHITINASE* and *BETA-1,2-GLUCANASE 3* coding for proteins with enzymatic activity against pathogens were up-regulated by compost. Taken together, these results suggest that enhanced expression of PR or related genes may explain increased plant resistance to *B. cinerea*. Interestingly, Zhang *et al.* [14] and Vallad *et al.* [13] described the involvement of PR proteins in the induction of resistance by compost. . *PHYTOALEXIN DEFICIENT 3*, which encodes CYP71B15 [30] that converts dihydrocamalexic acid to camalexin [31], and *CYTOCHROME P450 (CYP71A13)*, which is also involved in camalexin synthesis and is up-regulated by chitosan (a chitin derivative) treatment [32], were up-regulated in compost-grown plants. Camalexin shows cytotoxicity [33], particularly against eukaryotic pathogens. Thus, up-regulation of camalexin synthesis might also contribute to compost-induced resistance.

Several genes related to SAR were up-regulated in compost-grown plants, suggesting that compost-induced resistance shares similarities with this plant defense phenomenon. The *Arabidopsis* SA-response mutant *pbs3* disrupts *AVRPPHB SUSCEPTIBLE 3 (PBS3)*, resulting in enhanced susceptibility to *Pseudomonas syringae* infection due to SA signaling defects [34]. Over-expression of *CAM-BINDING PROTEIN 60 G-LIKE (CBP60G)* in Arabidopsis causes high SA accumulation, increased expression of defense genes and enhanced resistance to *Pseudomonas syringae*. Plants over-expressing *CBP60G* also show hypersensitivity to ABA and enhanced tolerance to drought stress. *CBP60G* serves as a molecular link that positively regulates ABA- and SA-mediated pathways in plants [35]. *SAR-DEFICIENT 1 (SARD1)* and *CBP60G* are key regulators for *ISOCHORISMATE SYNTHASE 1 (ICS1)* induction and SA synthesis. The involvement of SA signaling is also supported by the up-regulation of *ENHANCED DISEASE SUSCEPTIBILITY 5 (EDS5)*, which is required for SA synthesis in response to pathogen inoculation [36].

Composts are known to harbor billions of colony-forming units of micro-organisms per gram, while inert substrates, such as perlite, are naturally much less colonized [2]. The factors responsible for the induction of systemic resistance present in certain composts are heat-labile [13]. Along these lines, several micro-organism strains have been described as inducing either SAR or ISR in plants against a wide range of pathogens [10], [37]. It is very likely that the rich microbial populations present in the composts are responsible for this phenomenon. Some compost extracts also induce resistance even when sterilized, suggesting that the microbial component is not the only one capable of inducing resistance [38]. We found several genes relating to response to other organisms up-regulated in compost-grown plants. *FMO1* is required for full expression of TIR-NB-LRR-conditioned resistance to avirulent pathogens and for basal resistance to invasive virulent pathogens [39]. *AGD2-LIKE DEFENSE RESPONSE PROTEIN 1* (*ALD1*) is important for resistance to avirulent *P. syringae* strains, regulates camalexin accumulation and is essential for SAR [40]. *UDP-DEPENDENT GLYCOSYLTRANSFERASE 76B1 (UGT76B1)* over-expression leads to increased susceptibility to the biotrophic pathogen *Pseudomonas syringae* and increased resistance to necrotrophic *Alternaria brassicicola* [41]. The transcripts of *YELLOW-LEAF-SPECIFIC GENE 9* are accumulated during the hypersensitive response triggered with an avirulent *Cucumber mosaic virus* (CMV) strain [42]. *AVRRPT2-INDUCED GENE 1 (AIG1)* is involved in recognition of bacterial pathogens carrying the avirulence gene avrRpt2 [43]. These results suggest that the plant might perceive the compost as a source of incompatible pathogen interactions.

Pathogen recognition involves two kinds of receptors: those located in the plasma membrane and those present in the cytoplasm. Receptors located in the plasma membrane recognize conserved microbial patterns referred to as pathogen- or microbe-associated molecular patterns (PAMPs or MAMPs) and belong to families of receptor-like proteins (RLPs) and receptor-like kinases (RLKs), often with a leucine-rich repeat (LRR) [44]. Several RLPs, RLKs, LRR protein kinases and cysteine-rich RLKs were up-regulated in compost-grown plants, suggesting that compost elements might be recognized as PAMPs or MAMPs. Those pattern recognition receptors (PRRs) are the first layer of active plant immunity, as they respond to extracellular pathogen molecules before cellular invasion [45] and trigger several downstream responses, such as increase in cytosolic Ca^2+^, production of reactive oxygen species (ROS), activation of calcium-dependent and mitogen-activated protein kinases and reprogramming of gene transcription [46], including WRKY genes [47]. Increased levels of WRKY mRNA and protein and DNA-binding activity have been reported to be induced by infection with viruses, bacteria or oomycetes, by fungal elicitors, SA and wounding. WRKY proteins have a role in regulating subsequently activated secondary-response genes, whose products carry out protective and defensive reactions [47]. *WRKY38* and *WRKY40* were up-regulated by compost and are involved in SAR regulation and resistance to *B. cinerea* infection, respectively [48]. Virulent pathogens have acquired effectors that suppress PAMP-triggered immunity, resulting in effector-triggered susceptibility [49]. The second layer of active plant immunity is the recognition of these effectors by nucleotide binding (NB)-LRR type receptors in the cytosol [50]. This interaction is specific to plant cultivars and pathogen strains and is traditionally referred to as pathogen avirulence factors recognized by plant R genes [51]. As previously mentioned, the up-regulation of *ALD1*, *AIG1* and *FMO1*, which are related to avirulent pathogens, suggests that this second layer of pathogen recognition is also involved in compost-induced resistance.

Another major group of genes up-regulated by compost is of the genes related to salt, cold and water deprivation. Interestingly, it has been reported that a certain degree of salinity stress correlates with the ability of several composts to produce cucumber plants that are more resistant to *Botrytis cinerea* [17]. The transcription factor *DREB1A* was found to be up-regulated by compost. Over-expression of *DREB1A* improves stress tolerance to both freezing and dehydration in transgenic plants. In addition, *COR15a*, *COR 15b*, *COR78*, *GALACTINOL SYTHASE 3* and *LOW TEMPERATURE-INDUCED 30* are up-regulated in *DREB1A* over-expressor plants [52]. Interestingly, all these genes were also up-regulated by compost. *NAC DOMAIN CONTAINING 3* and *NAC DOMAIN CONTAINING 42*, which are involved in camalexin biosynthesis induction [53], as well as *MYB DOMAIN PROTEIN 47* whose expression is increased in response to JA and NaCl [54], were up-regulated by compost in our study. *RESPONSIVE TO ABA18 (RAB18)*, whose mRNA accumulates in plants exposed to low temperature, water stress or exogenous ABA [55], and *GALACTINOL SYNTHASE 2*, involved in the synthesis of oligosaccharides that function as osmoprotectants in plant cells [56], were up-regulated in compost-grown plants. The involvement of ABA in compost-induced gene expression is also supported by the up-regulation of *HIGHLY ABA-INDUCED PP2C GENE 2 (HAI2)*, a regulator of ABA signaling [57], and *PDR12*, which is a plasma membrane ABA uptake transporter [58].

Several genes involved in reduction and oxidation processes were found to be up-regulated by compost; indeed, oxireductase activity was the only molecular function-enriched GO term among the differentially expressed genes. ROS play a central role in plant defense against various pathogens [59]. They are directly toxic to pathogens [60] and can lead to a hypersensitive response, causing plant cell death and preventing further spread of biotrophic pathogens [61], [62]. ROS also serve as signals that lead to the activation of other defense mechanisms [63], [64]. During defense responses, ROS are produced by plant cells because of the enhanced enzymatic activities of plasma membrane-bound NADPH oxidases, cell wall-bound peroxidases (like *PEROXIDASE 37*, up-regulated by compost in the present study) and amine oxidases in the apoplast. ROS interact selectively with a target molecule that perceives the increased ROS concentration and then translates this information into a change of gene expression. Such a change in transcriptional activity may be achieved through the oxidation of components of signaling pathways that subsequently activate transcription factors or by modifying a redox-sensitive TF directly. Interestingly, during a SAR response, a change in cellular reduction potential occurs, resulting in the reduction of NON-EXPRESSOR OF PR1, an essential regulator of SAR, to a monomeric form that accumulates in the nucleus and activates gene expression [65]. The rapid generation of ROS is central to disease resistance responses and to ABA signaling [16]. Recent evidence suggests the existence of a significant overlap between signaling networks that control abiotic stress tolerance and disease resistance. Indeed, the above-mentioned *HAI2* is up-regulated by *Botrytis cinerea*, *Pseudomonas syringae* pv. *tomato*, oxidative stress, salinity, cold and drought, as well as ABA application [57].

In addition to SA- and ABA-related genes, it is worth mentioning that *CYTOKININ OXIDASE 4* and *GIBBERELLIN 2-OXIDASE 1*, which catalyze the inactivation of cytokinins and gibberellins, respectively [66]–[67], were up-regulated by compost, as was *AMINOCYCLOPROPANE-1-CARBOXYLATE SYNTHASE*, which catalyzes the conversion of S-adenosyl-methionine to ACC, the precursor of ethylene [68].

The enrichment of transcription factor binding sites observed in the up-regulated genes are related to ABA response (ABRE-like binding site motif) and cold and dehydration stress (DREB/CBF1). The W-box is the binding site of the above-mentioned WRKY transcription factors [47]. These results are consistent with the observed patterns of gene expression, particularly ABA-dependent and -independent stress responses and SA/SAR-mediated responses. In addition, enrichment of Evening Element promoter motif, related to the circadian clock, and CACGTG motif and Z-box promoter, related to light regulation, are also enriched in genes up-regulated by exogenous ABA treatments, suggesting a link between these regulatory elements and ABA [69].

We also studied the effect of *B. cinerea* inoculation on the expression of 10 genes affected by compost treatment. The objective was to answer the question of whether the genes enhanced by compost were the same as the plant used later on to defend itself against the pathogen. As shown in the results, some gene expression showed strengthening of the expression when compost-grown plants were inoculated with the pathogen and other genes were equally induced by compost or pathogen treatment, while others were less induced by the double treatment than compost alone. This broad range of behavior suggests that plants respond to compost treatment with a complex array of responses that may or may not be directly related to plant defense. It leaves the door open to hypothesizing whether this compost-induced resistance might be more effective against biotrophic pathogens or not, since these are counteracted by means of SA plant responses [70].

In conclusion, compost triggers a plant response that shares similarities with both SAR and ABA-dependent/independent abiotic stress responses. As expected, compost acts as both a biotic and abiotic stimulus. The plant responds to these stimuli as it will respond to bacteria, fungi, cold, water deprivation and oxidative stress. The defense responses triggered are in some way similar to those triggered by an incompatible interaction, with an up-regulation of the secondary metabolism and metabolism of aromatic compounds, in which the redox state is an important factor, as deduced from the importance of the oxireductase activities triggered by compost.

## Supporting Information

Table S1 **List of significant differentially expressed genes from Arabidopsis plants grown in compost, compared to plants grown in perlite (FC>1).**
(XLS)

Table S2 **List of transcription factor binding sites in the 1,000 bp upstream promoter sequences of significant differentially expressed genes.**
(XLS)
